# miR-375-3p inhibits the progression of laryngeal squamous cell carcinoma by targeting hepatocyte nuclear factor-1β

**DOI:** 10.3892/ol.2020.11941

**Published:** 2020-08-03

**Authors:** Kunpeng Chang, Zhenxing Wei, Hua Cao

**Affiliations:** 1 Department of Otolaryngology Head and Neck Surgery, Luoyang Central Hospital Affiliated to Zhengzhou University, Luoyang, Henan 471000, P.R. China; 2 Department of Otolaryngology Head and Neck Surgery, The First Affiliated Hospital of Zhengzhou University, Zhengzhou, Henan 450052, P.R. China

**Keywords:** microRNA-375-3p, laryngeal squamous cell carcinoma, hepatocyte nuclear factor-1β

## Abstract

Laryngeal squamous cell carcinoma (LSCC) is one of the most frequently diagnosed head and neck cancers worldwide. Increasing evidence suggests that microRNAs (miRNAs/miRs) regulate the progression of tumorigenesis and the malignant behaviors of cancer cells. The aim of this study was to investigate the function and underlying mechanism of miR-375-3p in LSCC. The expression of miR-375-3p in LSCC tissues and cells was detected using reverse transcription-quantitative PCR. The effects of miR-375-3p on the malignant phenotype of LSCC cells was determined using the Cell Counting Kit-8 assay and flow cytometry. The targets of miR-375-3p were predicted using the miRDB database and confirmed by the luciferase reporter assay. The results of the present study demonstrated that miR-375-3p was downregulated in LSCC tissues and cell lines. Furthermore, overexpression of miR-375-3p significantly suppressed the proliferation and cell cycle progression of LSCC cells. Overexpression of miR-375-3p also increased LSCC cell apoptosis. Mechanistical analysis indicated that miR-375-3p bound the 3′-untranslated region of the hepatocyte nuclear factor 1β (HNF1β) and decreased its expression in LSCC cells. Consistent with the role of HNF1β in glucose metabolism, overexpression of miR-375-3p significantly inhibited glucose consumption and lactate production in LSCC cells. Transfection with HNF1β notably reversed the inhibitory effect of miR-375-3p on the proliferation of LSCC cells. Collectively, these results indicate the tumor suppressive role of miR-375-3p in LSCC via HNF1β, suggesting that miR-375-3p may serve as a potential target in the treatment of LSCC.

## Introduction

Laryngeal squamous cell carcinoma (LSCC) is a common type of laryngeal cancer globally that frequently occurs in the larynx and accounts for 95% of laryngeal cancer cases in the recent decade (1–3). Surgical resection, chemotherapy, radiotherapy and immunotherapy have been used in the treatment of LSCC (4). However, the mortality rate of patients with advanced LSCC remains high due to the limitations of these therapeutic strategies, such as chemo- and radio-resistance (5). The 5-year overall survival rate of patients with LSCC is ~64% (5). In addition, some patients progress to advanced stages of the disease due to disease recurrence and metastasis (6). Thus, understanding the pathogenesis and identification of novel therapeutic targets are critical for the effective treatment of LSCC.

MicroRNAs (miRNAs/miRs) are a class of small non-coding RNA molecules that are ~22 nucleotides in length (7–9). miRNAs were primarily identified as key regulators of gene expression by binding the 3′-untranslated region (UTR) of target mRNAs (9). Increasing evidence suggests that miRNAs serve important roles across a variety of physiological and pathological conditions, including cell proliferation, apoptosis and cell cycle progression (10,11). Notably, the novel function of miRNAs as potential biomarkers and therapeutic targets in the treatment of different types of cancer is emerging (12–15). miRNAs negatively modulate the expression of cancer-associated genes, and thus regulate cancer progression (16,17). Aberrant expression of miRNAs has been reported in LSCC, and is associated with angiogenesis, tumor growth and metastasis (18–20). For example, miR-143-3p suppresses the proliferation and invasion of LSCC cells by targeting melanoma-associated antigen A9 (21). A recent study demonstrated that miR-154 inhibits the progression of LSCC by regulating N-acetylgalactosaminyltransferase 7 (GALNT7) (22). Furthermore, miR-4497 was identified as a tumor suppressor in LSCC that negatively modulates gastrulation brain homeobox 2 (GBX2) expression (23). Notably, a previous meta-analysis predicted the potential function and clinical significance of miR-375-3p in head and neck squamous cell carcinoma (HNSCC) (24). In this study, a total of 21 studies involving 1,685 subjects were analyzed to evaluate the relationship between miRNA and the prognosis of HNSCC (24). Significantly decreased expression of miR-375-3p was found in HNSCC and was associated with poor prognosis of patients (24). However, the underlying molecular mechanism of miR-375-3p in LSCC remains unknown.

Aerobic glycolysis, also known as the ‘Warburg effect’, is considered the primary metabolic process for cancer cells that facilitates cell proliferation under hypoxic conditions (25–27). The glycolysis of cancer cells is catalyzed by several key regulators, including glucose transporter 1 (GLUT1), lactate dehydrogenase A (LDHA) and hypoxia-inducible factor 1α (HIF1α) (26). Previous studies have reported that the hepatocyte nuclear factor 1β (HNF-1β), also known as TCF2, is a key transcription factor that promotes carcinogenesis by regulating glucose metabolism (28–30). Overexpression of HNF1β has been observed in different types of human cancer and is associated with the poor prognosis of patients with cancer (31–34). A previous study demonstrated that HNF1β is targeted by miRNAs and negatively modulated in cancer cells (35).

The aim of this study was to explore the function and mechanism of miR-375-3p in LSCC. The expression of miR-375-3p in LSCC tissues and cells was detected using reverse-transcription quantitative (RT-q)PCR. CCK-8 assay, cell apoptosis and cell cycle analysis were performed to evaluate the effects of miR-375-3p on the malignant behavior of LSCC cells. The results of the present study indicated that miR-375-3p expression decreased in LSCC tissues and cell lines. Furthermore, overexpression of miR-375-3p inhibited LSCC cell proliferation. Functional analysis demonstrated that miR-375-3p targeted HIF1β and inhibited its expression. Taken together, these results provide a novel insight by which miR-375/HNF1β signaling regulates the progression of LSCC.

## Materials and methods

### 

#### LSCC tissues, cell lines and plasmids

A total of 50 paired LSCC tissues and matched adjacent normal tissues (>5 cm from the margin of the LSCC tissues) were collected from patients (20 female and 30 male; age range, 39–74 years old; mean age, 58.5 years old) diagnosed with LSCC at the First Affiliated Hospital of Zhengzhou University between January 2012 to December 2014. Tissues were collected via surgical resection and confirmed via histopathological analysis by 3 independent pathologists from the First Affiliated Hospital of Zhengzhou University (Zhengzhou, China). Tissues were frozen in liquid nitrogen and stored at −80°C before further experiments. The present study was approved by the Ethical Institution of The First Affiliated Hospital of Zhengzhou University (Zhengzhou, China; approval no. 20120134-445) and performed in accordance with The Declaration of Helsinki. All patients provided written informed consent prior to the study start. Patients were divided into high and low miR-375-3p expression groups with a median cut-off value of 3.68.

The LSCC cell lines, AMC-HN-8, Tu-177 and Tu-212 (the Tu-212 cell line used in the present study was authenticated by STR profiling) were purchased from the American Type Culture Collection, while the human bronchial epithelial 16 HBE cell line (cat. no. scc150) was purchased from Sigma-Aldrich; Merck KGaA. Cells were maintained in DMEM (Thermo Fisher Scientific, Inc.) supplemented with 10% FBS (Invitrogen; Thermo Fisher Scientific, Inc.) at 37°C in 5% CO2.

The Flag-HIF1β plasmid was generated by amplifying the full-length of HIF1β via PCR using the cDNA from AMC-HN-8 cells with JumpStartTM Taq DNA polymerase (cat. no. D9307; Sigma-Aldrich; Merck KGaA). The thermocycling conditions used were as follows: initial denaturation at 95°C for 5 min followed by 33 cycles of denaturation at 95°C for 30 sec, annealing at 58°C for 30 sec, elongation at 72°C for 1 min and final extension at 72°C for 5 min. The PCR products were inserted into the backbone of the Flag-vector (Beijing Solarbio Science and Technology Co., Ltd) at the restriction enzyme sites of *Not*I and *Apa*I. The primers of HIF1β were designed as follows: forward, 5′-GCGGCGATGGCGGCGACTA-3′ and reverse, 5′-GGGCCCCTAGAGTTCCTGTTG-3′.

#### Transfection

Transfection of AMC-HN-8 and Tu-212 cells was performed using Lipofectamine 2000 reagent (Invitrogen; Thermo Fisher Scientific, Inc.). miR-375-3p mimic (5′-UUUGUUCGUUCGGCUCGCGUGA-3′), miR-375-3p inhibitor (5′-UCACGCGAGCCGAACGAACAAA-3′) and control miRNA (5′-GGUUCGUACGUACACUGUUCA-3′) were obtained from the Guangzhou RiboBio Co., Ltd. 50 nm of miRNA was diluted with 100 µl OPTI-MEM (Invitrogen; Thermo Fisher Scientific, Inc.) and subsequently incubated with 5 µl Lipofectamine 2000 for 15 min at room temperature. Subsequent experimentation was performed 48 h post-transfection.

#### RT-qPCR

Total RNA was extracted from LSCC tissues or cells using TRIzol regent (Invitrogen; Thermo Fisher Scientific, Inc.), and RNA concentration was measured using the NanoDrop 2000 spectrophotometer (Thermo Fisher Scientific, Inc.). RT was performed using the Revert Aid First Strand cDNA synthesis kit (Thermo Fisher Scientific, Inc.), with random primers at of 25°C for 5 min, followed by 42°C for 60 min and 70°C for 5 min. The level of miR-375-3p was assessed using the SYBR Green PCR Mixture (Applied Biosystems; Thermo Fisher Scientific, Inc.) and ABI Prism 7900 detection system (Bio-Rad Laboratories, Inc.). The following primer sequences were used for qPCR: miR-375-3p forward, 5′-CGGGTTTGTTCGTTCGGCT-3′ and reverse, 5′-GTGCAGGGTCCGAGGTATT-3′; U6 forward, 5′-GCTTCGGCAGCACATATACT-3′ and reverse, 5′-GTGCAGGGTCCGAGGTATTC-3′; HNF1β forward, 5′-ACACACCTCCCATCCTCAAG-3′ and reverse, 5′-CATTTTAGCAGCCCTCCAAG-3′; and GAPDH forward, 5′-AAATCCCATCACCATCTTCCAG-3′ and reverse, 5′-TGATGACCCTTTTGGCTCCC-3′. The following thermocycling conditions were used for qPCR: initial denaturation at 95°C for 2 min followed by 40 cycles of denaturation at 95°C for 15 sec, annealing and elongation at 60°C for 30 sec. Relative expression levels were calculated using the 2^−∆∆Cq^ method (36) and normalized to the internal reference genes U6 and GAPDH.

#### Cell proliferation assay

The proliferative rate of AMC-HN-8 and Tu-212 cells transfected with miR-375-3p mimics or inhibitor was determined using the cell counting Kit-8 (CCK-8) according to the manufacturer's instructions. Cells were seeded into 96-well plates at a density of 2,000 cells/well and cultured with Dulbecco's Modified Eagle's Medium (DMEM) at 37°C overnight. Subsequently, 10 µl CCK-8 solution (Dojindo Molecular Technologies, Inc.) was added into each well and further incubated for 4 h at 37°C. Cell proliferation was analyzed at a wavelength of 450 nm, using a microplate reader (Bio-Rad Laboratories, Inc.). All experiments were performed in triplicate.

#### Target prediction

Targets of miR-375-3p were predicted using the miRDB online database (http://mirdb.org/) by providing the name of miRNA as ‘miR-375-3p’.

#### Dual-luciferase reporter assay

AMC-HN-8 and Tu-212 cells were seeded into 96-well plates at a density of 1,000 cells/well and cultured with DEME medium at 37°C overnight. Cells were subsequently co-transfected with miR-375-3p mimic or inhibitor and the pmirGLO luciferase vector (Promega Corporation) containing wild type (WT) or mutant (MUT) 3′-UTR of HNF1β using Lipofectamine 2000 (Invitrogen; Thermo Fisher Scientific, Inc.). Following incubation for 48 h at 37°C, cells were harvested and firefly and *Renilla* luciferase activities were detected using a Dual-luciferase Reporter Assay system (Promega Corporation), according to the manufacturer's protocol. Firefly luciferase activity was normalized to *Renilla* luciferase activity. All experiments were performed in triplicate.

#### Western blotting

Total protein was extracted using RIPA lysis buffer (Beyotime Institute of Biotechnology) containing protease inhibitors and phosphatase inhibitors (Thermo Fisher Scientific, Inc.). Protein concentration was determined using a bicinchoninic acid (BCA) assay and an equal amount of protein (20 µg) was separated by 15% SDS-PAGE. The separated proteins were subsequently transferred onto PVDF western blotting membranes (Roche Diagnostics) and blocked with 5% skim milk for 1 h at room temperature. Membranes were washed twice with TBST (0.1% Tween-20) and incubated with primary antibodies against HNF1β (1:1,000 dilution; cat. no. ab236759; Abcam) and GAPDH (1:2,000 dilution; cat. no. 5174; Cell Signaling Technology, Inc.) overnight at 4°C. Membranes were re-washed twice with TBST (0.1% Tween-20) and incubated with goat anti-mouse (cat. no. 170-6516) or goat anti-rabbit (cat. no. 170-6515) IgG (H+L)-horseradish peroxidase-conjugated secondary antibodies (1:5,000 dilution; Bio-Rad Laboratories, Inc.) at room temperature for 1 h. Protein bands were visualized using the ECL Western Blotting Substrate (Thermo Fisher Scientific, Inc.), according to the manufacturer's protocol.

#### Flow cytometric analysis of apoptosis

AMC-HN-8 and Tu-212 cells were seeded into 6-well plates (1×10^5^ cells/well) and transfected with miR-375-3p mimics or inhibitor. After 48 h, cells were collected, washed twice with PBS and stained with Annexin V (Invitrogen; Thermo Fisher Scientific, Inc.) for 15 min in the dark at room temperature. Cells were subsequently stained with propidium iodide (PI) solution (Invitrogen; Thermo Fisher Scientific, Inc.) at room temperature for 1 min. The apoptotic cells were analyzed using a flow cytometer (BD FACSCalibur; BD Biosciences) and FlowJo software version 10.6 (FlowJo LLC).

#### Glucose uptake

AMC-HN-8 and Tu-212 cells transfected with miR-375-3p mimics or inhibitor were cultured in serum-free DMEM medium at 37°C overnight. Glucose uptake was determined using the Glucose Uptake Assay kit (Colorimetric, cat. no. ab136955; Abcam) according to the manufacturer's instructions. Briefly, cells (1×10^5^) were washed twice with PBS and maintained in KRPH (Krebs-Ringer phosphate HEPES)/2% BSA for 40 min at RT, prior to incubation with 10 µl 2-deoxyglucose (2-DG) for 15 min at room temperature. Standard 2-DG6P solutions (30 µl) or equal volumes of media for miR-375-3p mimic or miR-control transfected cells were added into 96-well plates, respectively. Subsequently, Assay buffer (8 µl) and the enzyme mix (2 µl) were added to the solution and incubated for 1 h at room temperature. Extraction buffer (90 µl) was added and heated at 90°C for 30 min, prior to incubation with reaction buffer B (35 µl) at room temperature for 5 min. Glucose uptake was analyzed at a wavelength of 412 nm, using a microplate reader (Bio-Rad Laboratories, Inc.). Total protein was quantified using the BCA kit (Beyotime Institute of Biotechnology) according to the manufacturer's protocol.

#### Lactate production

AMC-HN-8 and Tu-212 cells (1×10^5^) transfected with miR-375-3p mimics or inhibitor were seeded into 96-well plates and cultured with DMEM medium at 37°C. After 48 h, lactate production was measured using the Lactate Assay kit (cat. no. MAK064; Sigma-Aldrich; Merck KGaA), according to the manufacturer's protocol. Cells were lysed and centrifuged at 10,000 × g for 10 min at 4°C. The supernatant (50 µl) was transferred into the wells following deproteinization with a 10 kDa MWCO spin filter. The lactate standard was established by adding 0, 2, 4, 6, 8 and 10 µl of 1 nmol/μl lactate solution to the lactate assay buffer to make a final volume of 50 µl. Following incubation with 50 µl master reaction mix at room temperature for 30 min, lactate production was analyzed at a wavelength of 570 nm, using a microplate reader (Bio-Rad Laboratories, Inc.). Total protein was quantified for each group using the BCA kit (Beyotime Institute of Biotechnology), according to the manufacturer's protocol.

#### Statistical analysis

Statistical analysis was performed using GraphPad Prism software (version 7.0; GraphPad Software Inc.) and data are presented as the mean ± standard deviation. A paired Student's t-test was used to compare differences between two groups. One-way ANOVA followed by Tukey's post-hoc test were used to compare differences between multiple groups. The χ^2^ test was used to determine the association between miR-375-3p expression and clinicopathological characteristics of patients with LSCC. P<0.05 was considered to indicate a statistically significant difference.

## Results

### 

#### miR-375-3p is downregulated in LSCC tissues and cell lines

**Figure 1.**
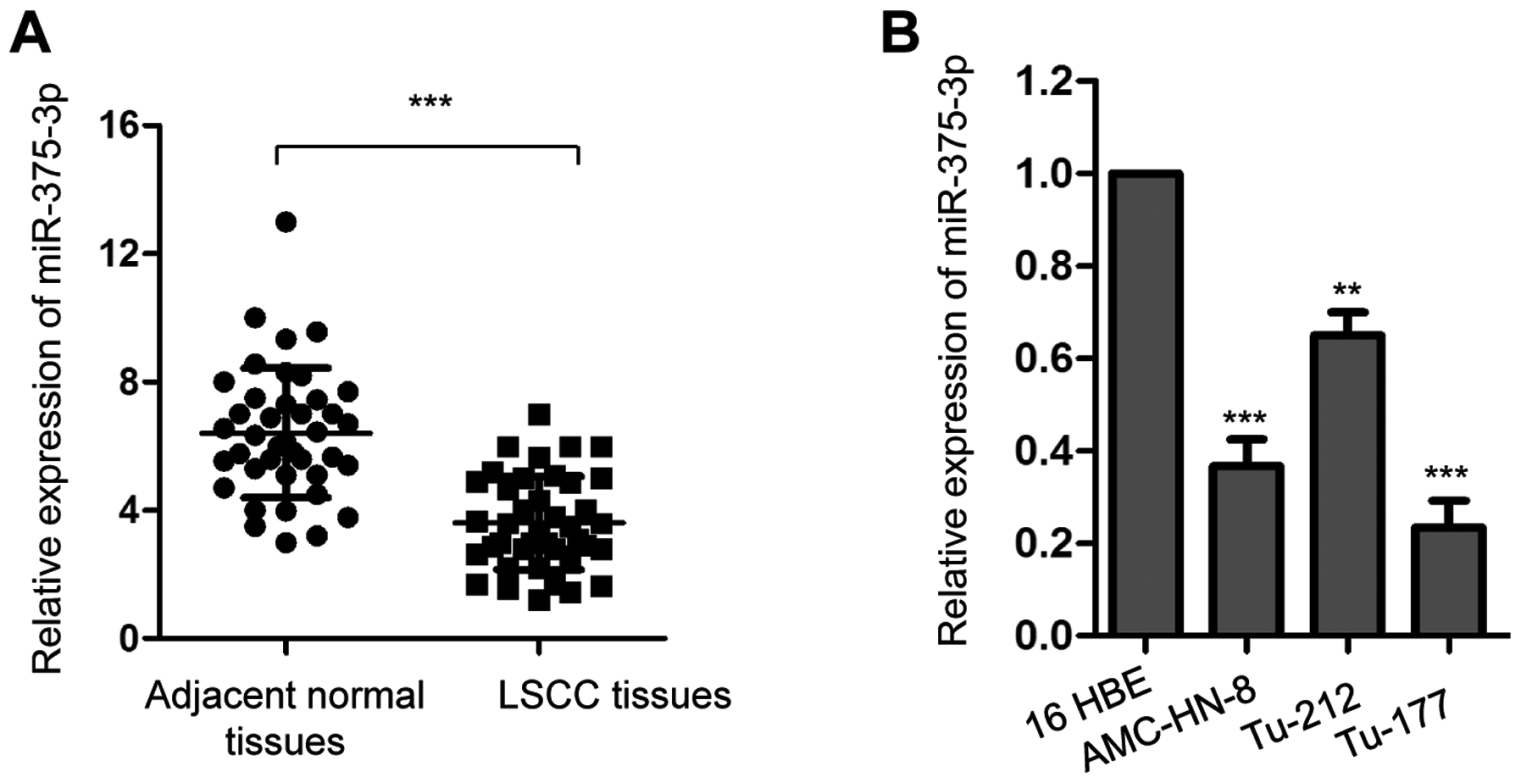
miR-375-3p expression is downregulated in LSCC. (A) Reverse transcription-quantitative PCR analysis was performed to assess miR-375-3p expression in 50-paired LSCC tissues and adjacent normal tissues. (B) miR-375-3p expression was compared between LSCC cell lines (AMC-HN-8, Tu-212 and Tu-177) and normal human bronchial epithelial 16 HBE cells. **P<0.01, ***P<0.001 vs. 16 HBE cells. miR, microRNA; LSCC, laryngeal squamous cell carcinoma.

RT-qPCR analysis was performed to assess miR-375-3p expression in 50 paired LSCC tissues and matched adjacent normal tissues. miR-375-3p expression was significantly downregulated in LSCC tissues compared with adjacent normal tissues (Fig. 1A; ***P<0.001). Consistently, miR-375-3p expression was significantly downregulated in the LSCC cell lines (AMC-HN-8, Tu-212 and Tu-177) compared with 16 HBE cells (Fig. 1B; ***P<0.001).

In order to further determine the clinical significance of miR-375-3p in LSCC, patients with LSCC were divided into low and high miR-375-3p expression groups, respectively, with a median miR-375-3p expression of 3.68 set as the cut-off value. The results demonstrated that miR-375-3p expression was significantly associated with tumor size, TNM stage (37), metastasis and histological grade (38) of patients with LSCC (Table I). Taken together, these results indicate the potential involvement of miR-375-3p in LSCC.

#### miR-375-3p regulates the proliferation and apoptosis of AMC-HN-8 and Tu-212 cells

**Figure 2.**
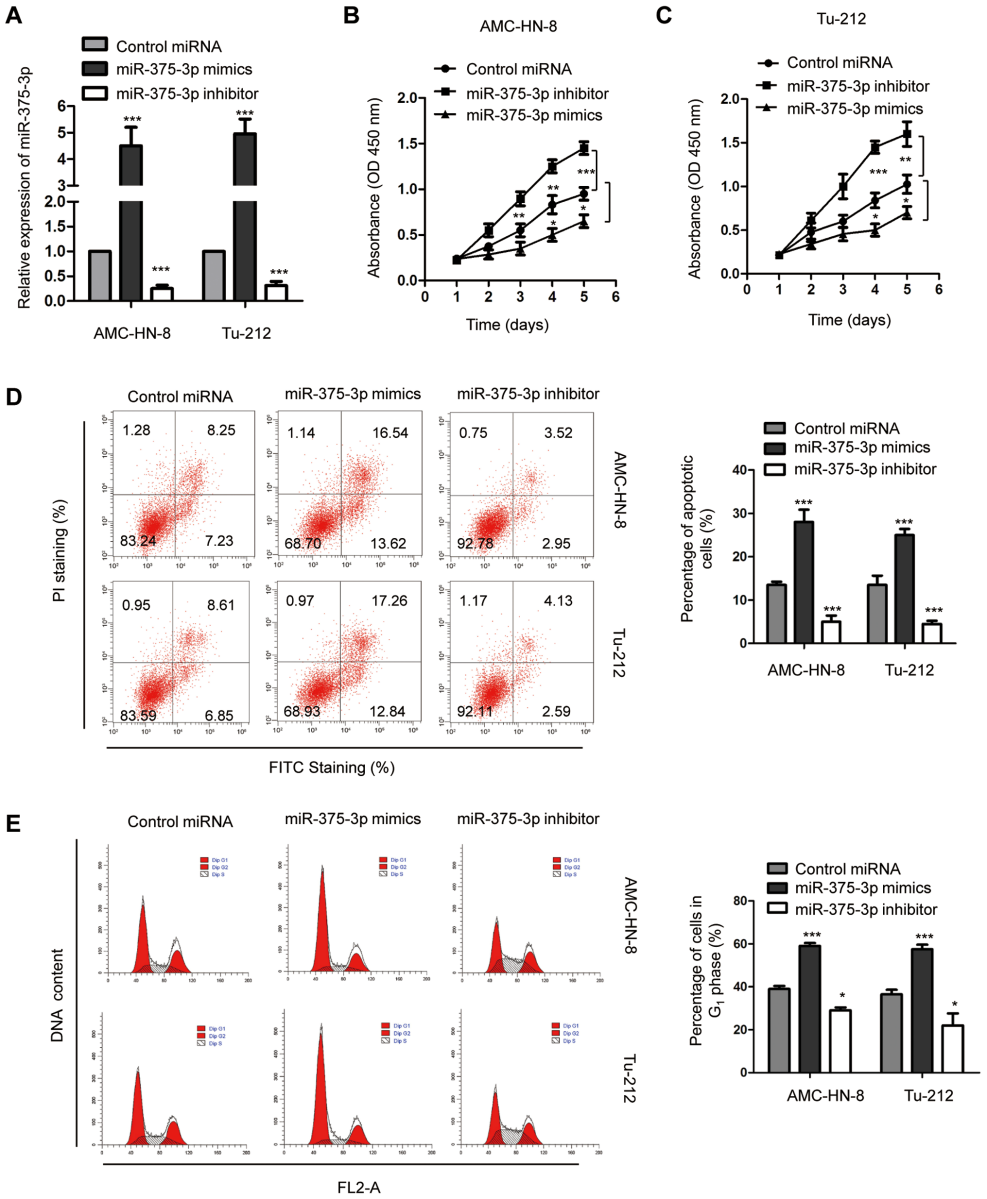
miR-375-3p regulates the proliferation of LSCC cells. (A) Reverse transcription-quantitative PCR analysis was performed to determine miR-375-3p expression in AMC-HN-8 and Tu-212 cells transfected with miR-375-3p mimics or inhibitor. Aberrant miR-375-3p expression modulated the proliferation of (B) AMC-HN-8 and (C) Tu-212 cells. (D) LSCC cells were transfected with miR-375-3p mimics or inhibitor and cell apoptosis was determined via flow cytometric analysis. (E) Cell cycle progression of LSCC cells was assessed following overexpression and depletion of miR-375-3p. *P<0.05 vs. control miRNA, ***P<0.001 vs. control miRNA. miR, microRNA; LSCC, laryngeal squamous cell carcinoma; PI, propidium iodide; OD, optical density; FITC, fluorescein isothiocyanate; FL2-A, fluorescence parameter 2-A.

In order to further investigate the biological function of miR-375-3p in LSCC, both AMC-HN-8 and Tu-212 cells, which are widely used in the study of LSCC (39), were transfected with miR-375-3p mimics or inhibitor, and transfection efficiency was determined via RT-qPCR analysis (Fig. 2A; ***P<0.001). The effect of miR-375-3p on the proliferative rate of AMC-HN-8 and Tu-212 cells was assessed via the CCK-8 assay. Overexpression of miR-375-3p significantly inhibited the proliferation of both AMC-HN-8 and Tu-212 cells, while miR-375-3p knockdown promoted the proliferation of AMC-HN-8 and Tu-212 cells (Fig. 2B and C; **P<0.01; ***P<0.001). Flow cytometric analysis was performed to detect the effect of miR-375-3p on the apoptosis of AMC-HN-8 and Tu-212 cells. The result demonstrated that apoptosis of both AMC-HN-8 and Tu-212 cells significantly increased following transfection with miR-375-3p mimics compared with the control cells (Fig. 2D; ***P<0.001). Conversely, miR-375-3p knockdown significantly decreased apoptosis of AMC-HN-8 and Tu-212 cells (Fig. 2D; ***P<0.001). Furthermore, overexpression of miR-375-3p significantly increased the proportion of cells in the G_0_/G_1_ phase (Fig. 2E; *P<0.05; ***P<0.001), suggesting that increased miR-375-3p expression induces G_1_ cell cycle arrest. miR-375-3p knockdown facilitated cell cycle progression from G_1_ to S phase (Fig. 2E; *P<0.05; ***P<0.001). Collectively, these results suggest the potential tumor suppressive role of miR-375-3p in regulating AMC-HN-8 and Tu-212 cell proliferation.

#### miR-375-3p targets HNF1β and decreases its expression in AMC-HN-8 and Tu-212 cells

**Figure 3.**
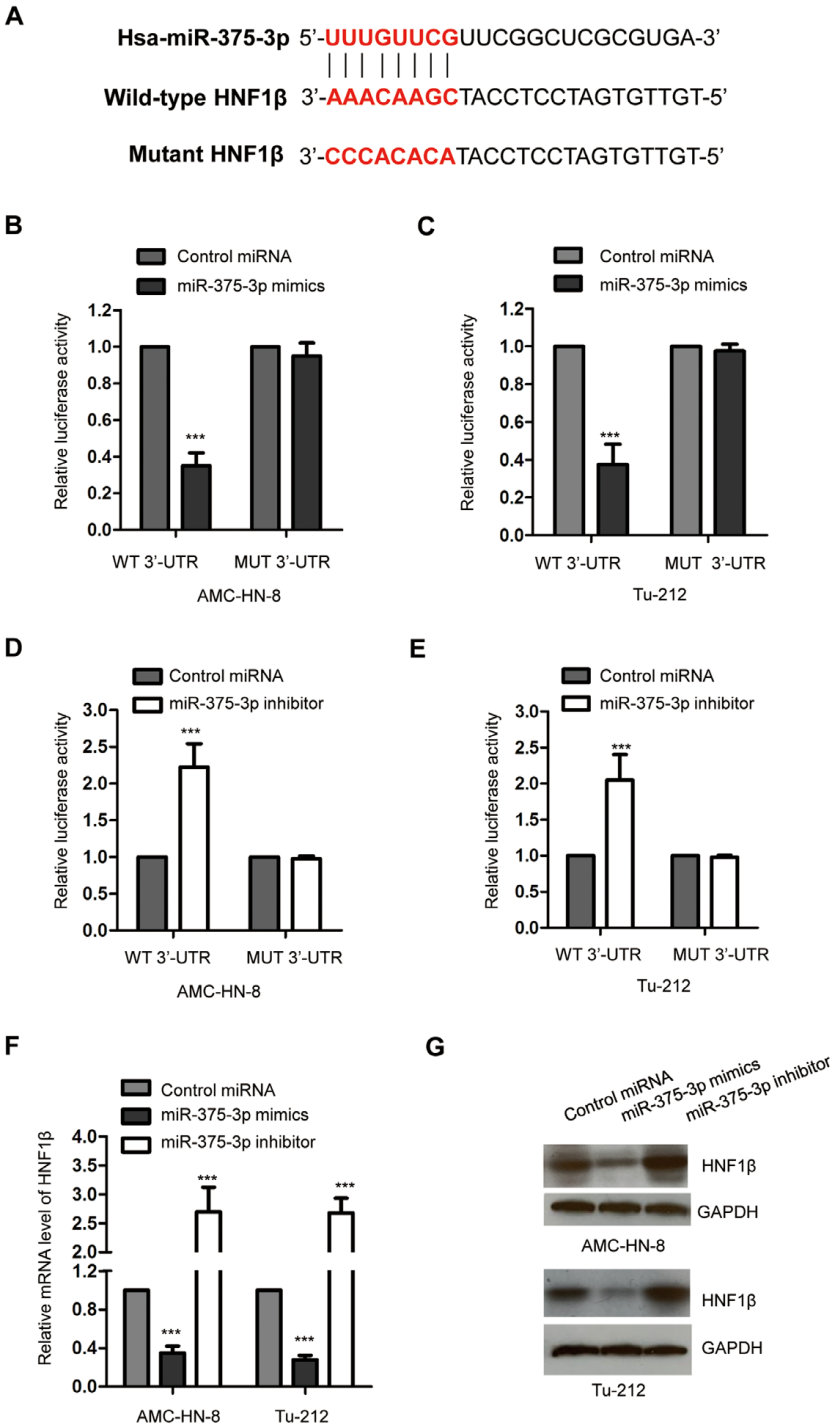
HNF1β is a target of miR-375-3p in LSCC cells. (A) The predicted binding sites of miR-375-3p at the 3′-UTR of HNF1β. Overexpression of miR-375-3p decreased the luciferase activity of (B) AMC-HN-8 and (C) Tu-212 cells expressing WT but not MUT 3′-UTR of HNF1β. miR-375-3p knockdown increased the luciferase activity of 3′-UTR of HNF1β in (D) AMC-HN-8 and (E) Tu-212 cells. (F) Overexpression of miR-375-3p inhibited the mRNA level of HNF1β, while miR-375-3p knockdown increased mRNA expression of HNF1β in LSCC cells. (G) Overexpression of miR-375-3p reduced the protein level of HNF1β and depletion of miR-375-3p increased the protein abundance of HNF1β. ***P<0.001 vs. control miRNA. HNF1β, hepatocyte nuclear factor 1β; miR, microRNA; LSCC, laryngeal squamous cell carcinoma; UTR, untranslated region; WT, wild-type; MUT, mutant.

The potential targets of miR-375-3p were predicted using the online miRDB database, in order to better understand the molecular mechanism by which miR-375-3p negatively regulates the progression of LSCC. The results indicated that the 3′-UTR of HNF1β contains a putative complementary binding site of miR-375-3p (Fig. 3A). In order to verify that HNF1β is a target of miR-375-3p, both AMC-HN-8 and Tu-212 cells were transfected with miR-375-3p mimics and the luciferase reporter vector harboring WT or MUT 3′-UTR of HNF1β. The results demonstrated that overexpression of miR-375-3p significantly decreased the luciferase activity of cells expressing the WT but not the MUT 3′-UTR of HNF1β (Fig. 3B and C; ***P<0.001). This indicated the specific binding between miR-375-3p and the 3′-UTR of HNF1β. In order to confirm these results, both AMC-HN-8 and Tu-212 cells were transfected with miR-375-3p inhibitor to decrease miR-375-3p expression. The dual-luciferase reporter assay demonstrated that decreased miR-375-3p expression significantly increased the luciferase activity of cells harboring WT 3′-UTR of HNF1β (Fig. 3D and E; ***P<0.001). Taken together, these results confirm the binding between miR-375-3p and the 3′-UTR of HNF1β.

RT-qPCR analysis was performed to determine whether the binding of miR-375-3p affected the stability of HNF1β mRNA in AMC-HN-8 and Tu-212 cells transfected with miR-375-3p mimics or inhibitor. The results demonstrated that overexpression of miR-375-3p significantly decreased HNF1β mRNA expression, while miR-375-3p knockdown significantly increased HNF1β mRNA expression in AMC-HN-8 and Tu-212 cells compared with cells expressing control-miRNA, respectively (Fig. 3F; ***P<0.001). Western blot analysis was performed to assess HNF1β protein levels in AMC-HN-8 and Tu-212 cells transfected with miR-375-3p mimics or inhibitor. The results indicated that overexpression of miR-375-3p decreased HNF1β protein expression, while miR-375-3p knockdown increased HNF1β protein expression in both AMC-HN-8 and Tu-212 cells compared with cells expressing control-miRNA, respectively (Fig. 3G). Collectively, these results suggest that miR-375-3p targets HNF1β and decreases it expression in LSCC cells.

#### miR-375-3p negatively regulates the glucose metabolism of AMC-HN-8 and Tu-212 cells by targeting HNF1β

**Figure 4.**
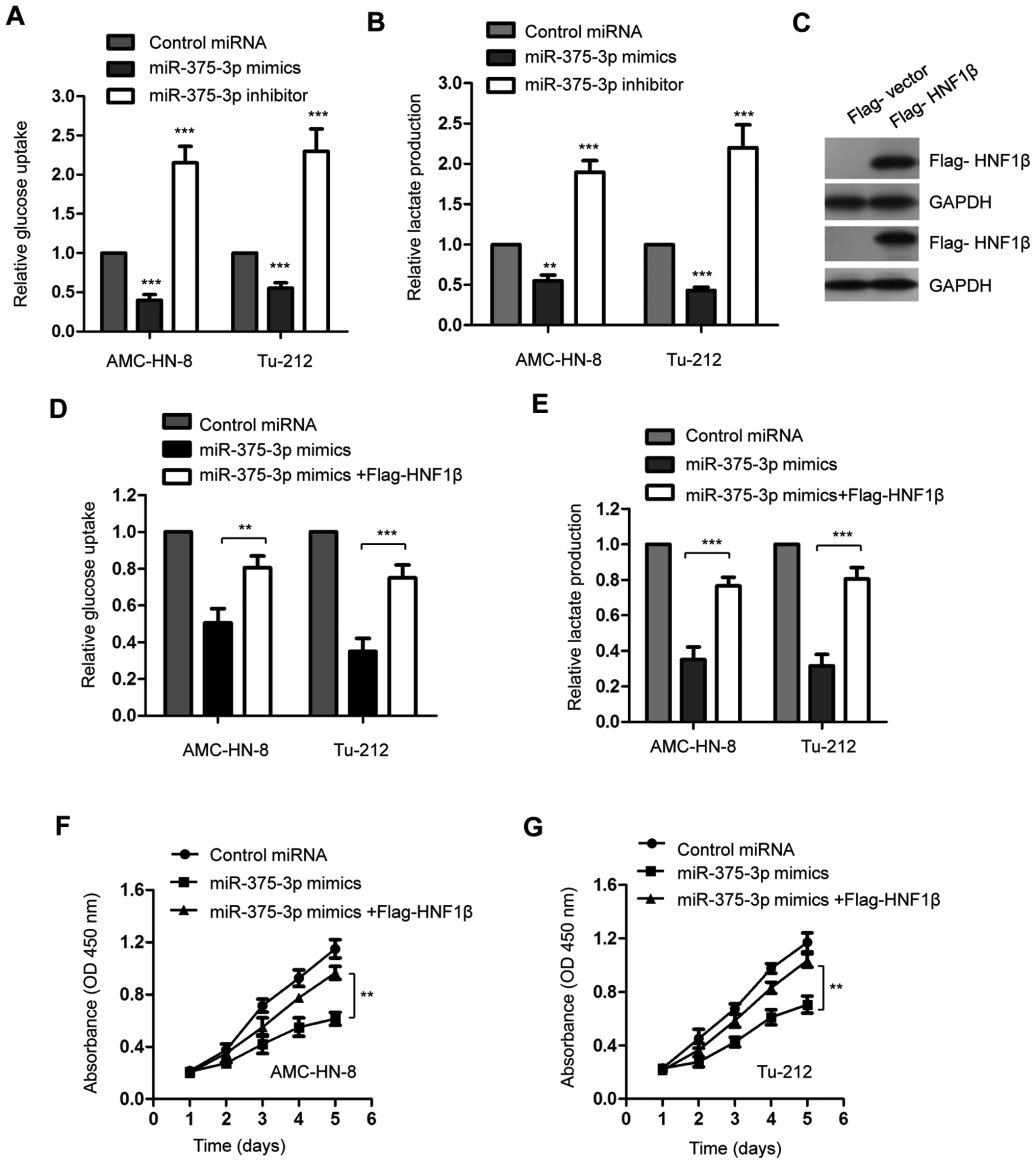
miR-375-3p regulates glucose metabolism of LSCC cells. (A) Overexpression of miR-375-3p decreased the glucose uptake of LSCC cells, while miR-375-3p knockdown increased glucose metabolism of LSCC cells. (B) Overexpressed miR-375-3p decreased the lactate production and inhibition of miR-375-3p increased the lactate generation of LSCC cells. (C) Western blot analysis was performed to assess HNF1β protein expression in AMC-HN-8 and Tu-212 cells transfected with Flag-vector or Flag-HNF1β. (D) Restoration of HNF1β significantly reversed the suppressive role of miR-375-3p in the glucose consumption LSCC cells. (E) Transfection of HNF1β attenuated the decreased lactate production of LSCC cells induced by miR-375-3p. (F) Overexpression of HNF1β significantly reversed the inhibitory effect of miR-375-3p on the proliferation of AMC-HN-8 cells. (G) Restoration of HNF1β reversed the suppressive effect of miR-375-3p on the proliferation of Tu-212 cells. **P<0.01 and ***P<0.001 vs. control miRNA. miR, microRNA; LSCC, laryngeal squamous cell carcinoma; HNF1β, hepatocyte nuclear factor 1β; OD, optical density.

Given that miR-375-3p decreased HNF1β expression in LSCC cells, the glucose uptake and lactate production of AMC-HN-8 and Tu-212 cells transfected with miR-375-3p mimics or inhibitor were assessed to determine the influence of miR-375-3p on the glucose metabolism of LSCC. The results indicated that overexpression of miR-375-3p significantly suppressed glucose consumption and lactate production in AMC-HN-8 and Tu-212 cells (Fig. 4A and B; **P<0.01; ***P<0.001). Consistently, miR-375-3p knockdown significantly promoted glucose metabolism of both AMC-HN-8 and Tu-212 cells (Fig. 4A and B; **P<0.01; ***P<0.001).

In order to confirm the involvement of HNF1β in suppressing glucose metabolism via miR-375-3p, HNF1β expression was rescued by transfecting LSCC cells with Flag-HNF1β. A vector encoding a FLAG tag was used to validate transfection efficiency of HIFIβ (data not shown). Transfection with Flag-HNF1β was validated via western blot analysis (Fig. 4C). Restoration of HNF1β significantly attenuated the inhibitory role of miR-375-3p in the glucose uptake and lactate generation of AMC-HN-8 and Tu-212 cells (Fig. 4D and E; **P<0.01; ***P<0.001).

Consistent with the important role of HNF1β in miR-375-3p-mediated suppression of glucose metabolism, overexpression of HNF1β significantly reversed the inhibitory effect of miR-375-3p on the proliferation of AMC-HN-8 and Tu-212 cells (Fig. 4F and G; **P<0.01). Taken together, these results suggest that miR-375-3p targets HNF1β to regulate glucose metabolism of AMC-HN-8 and Tu-212 cells.

## Discussion

The prognosis and treatment of LSCC have improved with advancements in therapeutic strategies; the 5-year overall survival rate of patients with LSCC is ~64% (5). Increasing evidence suggest that miRNAs exert tumor suppressive or oncogenic functions in the progression of LSCC (21,40–42). A previous study reported that miR-375-3p is downregulated in head and neck cancer (24). The results of the present study demonstrated that miR-375-3p expression was downregulated in LSCC tissues and cell lines, which was significantly associated with larger tumor size, higher pathological grades and distant metastasis of patients with LSCC. Taken together, these results suggest the tumor suppressive role of miR-375-3p in LSCC. However, further studies are required to investigate the expression and function of miR-375-3p in different types of cancer.

Due to the critical role miRNAs play in the progression of malignancy, they are considered promising targets in anticancer therapy (11,15,43). Consistent with the results following miR-375-3p knockdown in LSCC, overexpression of miR-375-3p inhibited the proliferation of LSCC cells. Furthermore, overexpression of miR-375-3p increased the apoptotic rate and induced cell cycle arrest at the G_1_ phase. Collectively, these results indicate that downregulation of miR-375-3p may serve as a promising biomarker for the prognosis of patients with LSCC, suggesting the role of miR-375-3p as a potential therapeutic target for the intervention of LSCC. In order to confirm the tumor suppressive role of miR-375-3p in LSCC, prospective studies will focus on investigating the effect of miR-375-3p on the growth of LSCC *in vivo*.

Given that miRNAs predominantly rely on modulating the expression of target genes, bioinformatics analysis was performed to identify the 3′-UTR of HNF1β containing the binding site of miR-375-3p, in order to determine the molecular mechanism underlying the suppressive function of miR-375-3p in LSCC. HNF1β is a transcription factor that plays a critical role during several processes, including cell proliferation, apoptosis and glucose metabolism (30,34). In the present study, overexpression of miR-375-3p significantly decreased the luciferase activity of cells expressing 3′-UTR of HNF1β. Consistently, transfection with miR-375-3p mimics inhibited mRNA and protein expression levels of HNF1β in LSCC cells. Notably, overexpression of HNF1β significantly reversed the inhibitory effect of miR-375-3p on the proliferation of LSCC cells. Collectively, these results suggest that HNF1β is a target of miR-375-3p, which mediates the suppressive role of miR-375-3p in LSCC. Given the results of the present study, the function of the miR-375-3p/HNF1β axis deserves further investigation in different types of cancer. Notably, a single miRNA has multiple targets (7,8), thus other targets of miR-375-3p may play key roles in the progression of LSCC.

In conclusion, the results of the present study demonstrated that miR-375-3p expression was downregulated in LSCC, which was associated with a poor prognosis. Furthermore, miR-375-3p targeted HNF1β and negatively regulated the proliferation of LSCC cells. Studies with larger numbers of patients with LSCC may be required to evaluate the clinical significance of miR-375-3p in the diagnosis and prognosis of LSCC. Additionally, *in vivo* studies are required to fully understand the tumor suppressive role of miR-375-3p in the progression of LSCC. Taken together, these results provide a novel insight into the pathogenesis of LSCC, suggesting that the miR-375-3p/HNF1β axis may function as a valuable therapeutic target for the treatment of LSCC.

## Figures and Tables

**Table I.** Association between miR-375-3p expression and clinicopathological characteristics of patients with laryngeal squamous cell carcinoma (n=50).

Characteristics	Number of patients, n	High miR-375-3p expression, n	Low miR-375-3p expression, n	P-value
Age, years				0.571
≤60	15	5	10	
>60	35	10	25	
Sex				0.632
Male	26	8	18	
Female	24	7	17	
Tumor size, cm				<0.001^a^
≤4	25	13	12	
>4	25	2	23	
Lymph node metastasis				<0.001^a^
Negative	22	10	12	
Positive	28	5	23	
Histological grade				<0.001^a^
High	30	10	20	
Poor	20	5	15	
TNM stage				<0.001^a^
I–II	26	11	15	
III–IV	24	4	20	

a P<0.001. miR, microRNA; TNM, tumor-node-metastasis.

## Data Availability

The datasets used and/or analyzed during the present study are available from the corresponding author upon reasonable request.

## References

[1] Lu ZM, Lin YF, Jiang L, Chen LS, Luo XN, Song XH, Chen SH, Zhang SY (2014). Micro-ribonucleic acid expression profiling and bioinformatic target gene analyses in laryngeal carcinoma. OncoTargets Ther.

[2] Tataru D, Mak V, Simo R, Davies EA, Gallagher JE (2017). Trends in the epidemiology of head and neck cancer in London. Clin Otolaryngol.

[3] Gao C, Hu S (2019). miR-506 is a YAP1-dependent tumor suppressor in laryngeal squamous cell carcinoma. Cancer Biol Ther.

[4] Marur S, Forastiere AA (2016). Head and Neck Squamous Cell Carcinoma: Update on Epidemiology, Diagnosis, and Treatment. Mayo Clin Proc.

[5] Palumbo A, De Martino M, Esposito F, Fraggetta F, Neto PN, Valverde Fernandes P, Santos IC, Dias FL, Nasciutti LE, Meireles Da Costa N (2018). HMGA2, but not HMGA1, is overexpressed in human larynx carcinomas. Histopathology.

[6] Ozmen OA, Alpay M, Saraydaroglu O, Demir UL, Kasapoglu F, Coskun HH, Basut OI (2018). Prognostic significance of soft tissue deposits in laryngeal carcinoma. Braz J Otorhinolaryngol.

[7] Ambros V (2004). The functions of animal microRNAs. Nature.

[8] Bartel DP (2004). MicroRNAs: Genomics, biogenesis, mechanism, and function. Cell.

[9] Fabian MR, Sonenberg N, Filipowicz W (2010). Regulation of mRNA translation and stability by microRNAs. Annu Rev Biochem.

[10] Mohr AM, Mott JL (2015). Overview of microRNA biology. Semin Liver Dis.

[11] Gentilin E, Degli Uberti E, Zatelli MC (2016). Strategies to use microRNAs as therapeutic targets. Best Pract Res Clin Endocrinol Metab.

[12] Momtazi AA, Shahabipour F, Khatibi S, Johnston TP, Pirro M, Sahebkar A (2016). Curcumin as a MicroRNA Regulator in Cancer: A Review. Rev Physiol Biochem Pharmacol.

[13] Iorio MV, Croce CM (2017). MicroRNA dysregulation in cancer: Diagnostics, monitoring and therapeutics. A comprehensive review. EMBO Mol Med.

[14] Asadzadeh Z, Mansoori B, Mohammadi A, Aghajani M, Haji-Asgarzadeh K, Safarzadeh E, Mokhtarzadeh A, Duijf PHG, Baradaran B (2019). microRNAs in cancer stem cells: Biology, pathways, and therapeutic opportunities. J Cell Physiol.

[15] Hosseinahli N, Aghapour M, Duijf PHG, Baradaran B (2018). Treating cancer with microRNA replacement therapy: A literature review. J Cell Physiol.

[16] Kwak PB, Iwasaki S, Tomari Y (2010). The microRNA pathway and cancer. Cancer Sci.

[17] Farazi TA, Spitzer JI, Morozov P, Tuschl T (2011). miRNAs in human cancer. J Pathol.

[18] Li X, Wang HL, Peng X, Zhou HF, Wang X (2012). miR-1297 mediates PTEN expression and contributes to cell progression in LSCC. Biochem Biophys Res Commun.

[19] Yungang W, Xiaoyu L, Pang T, Wenming L, Pan X (2014). miR-370 targeted FoxM1 functions as a tumor suppressor in laryngeal squamous cell carcinoma (LSCC). Biomed Pharmacother.

[20] Luo M, Sun G, Sun JW (2019). miR-196b affects the progression and prognosis of human LSCC through targeting PCDH-17. Auris Nasus Larynx.

[21] Han L, Tang M, Xu X, Jiang B, Wei Y, Qian H, Lu X (2018). miR-143-3p suppresses cell proliferation, migration, and invasion by targeting melanoma-associated antigen A9 in laryngeal squamous cell carcinoma. J Cell Biochem.

[22] Niu JT, Zhang LJ, Huang YW, Li C, Jiang N, Niu YJ (2018). miR-154 inhibits the growth of laryngeal squamous cell carcinoma by targeting GALNT7. Biochem Cell Biol.

[23] Chen X, Zhang L, Tang S (2019). MicroRNA-4497 functions as a tumor suppressor in laryngeal squamous cell carcinoma via negatively modulation the GBX2. Auris Nasus Larynx.

[24] Jamali Z, Asl Aminabadi N, Attaran R, Pournagiazar F, Ghertasi Oskouei S, Ahmadpour F (2015). MicroRNAs as prognostic molecular signatures in human head and neck squamous cell carcinoma: A systematic review and meta-analysis. Oral Oncol.

[25] Zheng J (2012). Energy metabolism of cancer: Glycolysis versus oxidative phosphorylation (Review). Oncol Lett.

[26] Akram M (2013). Mini-review on glycolysis and cancer. J Cancer Educ.

[27] Li XB, Gu JD, Zhou QH (2015). Review of aerobic glycolysis and its key enzymes - new targets for lung cancer therapy. Thorac Cancer.

[28] Amano Y, Mandai M, Yamaguchi K, Matsumura N, Kharma B, Baba T, Abiko K, Hamanishi J, Yoshioka Y, Konishi I (2015). Metabolic alterations caused by HNF1β expression in ovarian clear cell carcinoma contribute to cell survival. Oncotarget.

[29] Mandai M, Amano Y, Yamaguchi K, Matsumura N, Baba T, Konishi I (2015). Ovarian clear cell carcinoma meets metabolism; HNF-1β confers survival benefits through the Warburg effect and ROS reduction. Oncotarget.

[30] Okamoto T, Mandai M, Matsumura N, Yamaguchi K, Kondoh H, Amano Y, Baba T, Hamanishi J, Abiko K, Kosaka K (2015). Hepatocyte nuclear factor-1β (HNF-1β) promotes glucose uptake and glycolytic activity in ovarian clear cell carcinoma. Mol Carcinog.

[31] Kato N, Motoyama T (2009). Hepatocyte nuclear factor-1beta (HNF-1beta) in human urogenital organs: Its expression and role in embryogenesis and tumorigenesis. Histol Histopathol.

[32] Kobayashi H, Yamada Y, Kanayama S, Furukawa N, Noguchi T, Haruta S, Yoshida S, Sakata M, Sado T, Oi H (2009). The role of hepatocyte nuclear factor-1beta in the pathogenesis of clear cell carcinoma of the ovary. Int J Gynecol Cancer.

[33] Bockenhauer D, Jaureguiberry G (2016). HNF1B-associated clinical phenotypes: The kidney and beyond. Pediatr Nephrol.

[34] Bártů M, Dundr P, Němejcová K, Tichá I, Hojný H, Hájková N (2018). The Role of HNF1B in Tumorigenesis of Solid Tumours: A Review of Current Knowledge. Folia Biol (Praha).

[35] Zheng J, Liu X, Xue Y, Gong W, Ma J, Xi Z, Que Z, Liu Y (2017). TTBK2 circular RNA promotes glioma malignancy by regulating miR-217/HNF1β/Derlin-1 pathway. J Hematol Oncol.

[36] Livak KJ, Schmittgen TD (2001). Analysis of relative gene expression data using real-time quantitative PCR and the 2(-Delta Delta C(T)) method. Methods.

[37] Edge SB, Compton CC (2010). The American Joint Committee on Cancer: the 7th edition of the AJCC cancer staging manual and the future of TNM. Ann Surgl Oncol.

[38] Voigt JJ (1999). Recommendations of the FNCLCC Sarcoma Group for pathologic management of soft tissue sarcoma in adults. Ann Pathol.

[39] Shen Z, Yuan J, Tong Q, Hao W, Deng H, Li Q, Zhou C, Hu Y, Xu J (2020). Long non-coding RNA AC023794.4-201 exerts a tumor-suppressive function in laryngeal squamous cell cancer and may serve as a potential prognostic biomarker. Oncol Lett.

[40] Fan Y, Xia X, Zhu Y, Diao W, Zhu X, Gao Z, Chen X (2018). Circular RNA expression profile in laryngeal squamous cell carcinoma revealed by microarray. Cell Physiol Biochem.

[41] Yu CH, Xing FY, Zhang JY, Xu JQ, Li YC (2018). A combination of mRNA expression profile and miRNA expression profile identifies detection biomarkers in different tumor stages of laryngeal squamous cell carcinoma. Eur Rev Med Pharmacol Sci.

[42] Zhang F, Cao H (2019). MicroRNA-143-3p suppresses cell growth and invasion in laryngeal squamous cell carcinoma via targeting the k-Ras/Raf/MEK/ERK signaling pathway. Int J Oncol.

[43] Kaboli PJ, Rahmat A, Ismail P, Ling KH (2015). MicroRNA-based therapy and breast cancer: A comprehensive review of novel therapeutic strategies from diagnosis to treatment. Pharmacol Res.

